# Polymorphisms of T helper cell cytokine-associated genes and survival of hemodialysis patients – a prospective study

**DOI:** 10.1186/s12882-017-0582-x

**Published:** 2017-05-19

**Authors:** Alicja E. Grzegorzewska, Monika K. Świderska, Adrianna Mostowska, Wojciech Warchoł, Paweł P. Jagodziński

**Affiliations:** 1 Chair and Department of Nephrology, Transplantology and Internal Diseases, Poznan University of Medical Sciences, Przybyszewskiego 49, 60-355 Poznań, Poland; 2 Student Nephrology Research Group, Chair and Department of Nephrology, Transplantology and Internal Diseases, Poznan University of Medical Sciences, 5, Poznań, Poland; 3 Chair and Department of Biochemistry and Molecular Biology, Poznan University of Medical Sciences, Poznań, Poland; 4 Chair and Department of Biophysics, Poznan University of Medical Sciences, Poznań, Poland

**Keywords:** Cytokines, Genes, Hemodialysis, Interferon λ3, Survival, T helper cells

## Abstract

**Background:**

Circulating pro-inflammatory cytokines were associated with increased relative mortality risk, while immune parameters reflecting improved T-cell function were predictors of survival in hemodialysis (HD) patients. We evaluated in the prospective study whether variants in T helper cell cytokine-associated genes are determinants of mortality in HD patients.

**Methods:**

The study was carried out in 532 prevalent HD subjects who were followed-up for 7 years. HRM analysis was used for *IFNL3*, *IL12A*, *IL13*, and *IL4R* genotyping. *CCL2*, *IL12B*, and *IL18* were genotyped using PCR–RFLP analysis. Survival analyses were conducted using the Kaplan-Meier method and the Cox proportional hazard model.

**Results:**

In univariate analyses, *IFNL3* rs8099917 was associated with all-cause mortality in recessive model of inheritance (log-rank test *P* = 0.044), *IL12A* rs568408 - in dominant model (log-rank test *P* = 0.029). Minor homozygotes (the genotype GG) in *IFNL3* rs8099917 showed shorter survival during the study (3.6, 1.0–7.0 years vs 4.7, 0.1–7.0 years, *P* = 0.009) than the major allele (T) bearers. The rs8099917 GG patients demonstrated higher risk of death than the remaining patients (GT + TT) (OR 1.94, 95%CI 1.11–3.40, *P* = 0.020). Major homozygosity (the genotype GG) in *IL12A* rs568408 was associated with higher mortality than that shown in bearers of the minor allele (AA + AG) (HR 1.31, 95%CI 1.02–1.69, *P* = 0.035). In multivariate analyses, however, the mentioned polymorphisms were not independent predictors of survival.

**Conclusions:**

Polymorphisms of *IFNL3* rs8099917 and *IL12A* rs568408 contribute to survival of HD patients, but not as independent factors.

**Electronic supplementary material:**

The online version of this article (doi:10.1186/s12882-017-0582-x) contains supplementary material, which is available to authorized users.

## Background

Cardiovascular diseases, infections and cancers are the most common causes of death in hemodialysis (HD) patients.

After an almost 3-year mean follow-up period, higher levels of circulating pro-inflammatory cytokines were significantly associated with increased relative mortality risk, while immune parameters reflecting improved T-cell function were associated with survival in HD patients, independent of other medical risk factors [1]. Interleukin (IL)-6 and anti−/pro-inflammatory cytokine balance expressed as (IL-4 + IL-10)/IL-6 ratio were associated with an enhanced hazard ratio of cardiovascular mortality in HD patients [2]. Functional imbalance between regulatory T cells and T helper (Th) cells was also found as a contributor to the high incidence of cardiovascular events in this group [3]. Th1/Th2 cytokine balance evaluated by [IL-4 + IL-6 + IL-10)/(IL-2 + interferon (IFN)-γ] ratio was associated with non-cardiovascular (infection, malnutrition/decline, and neoplasm) mortality in HD patients [2]. A shift toward Th2 cells was demonstrated as very important in the carcinogenesis, and increased levels of Th2 cytokines are proposed as an early marker of cancer presence in the general population [4].

Polymorphisms of genes encoding Th1/Th2 cytokines were already associated with inflammatory response [5, 6], hypertension [7], atherosclerosis [5, 7], cardiovascular disease, comorbidity scores, functional scores, and biological/nutritional markers [8] in dialysis patients. However, data concerning associations between polymorphisms of Th cell cytokine-associated genes in respect to survival of HD patients are scarce. There were no demonstrable associations between alleles/genotypes and combinations of genotypes of IL-6, tumor necrosis factor-alpha, and IL-10 and mortality of HD patients in the HEMO Study [8]. In our retrospective study, the *IL13* rs20541 T allele and *IFNL3* rs8099917 GG genotype were negative predictors of survival in patients requiring renal replacement therapy, while the *IFNL3* rs12979860 TT genotype increased the risk of death only in patients negative for hepatitis B virus (HBV) or hepatitis C virus (HCV) infections [9]. Retrospective studies have drawbacks, including biases in the selection of patients. Therefore, our aim was to evaluate in the 7-year prospective study whether variants in selected Th cell cytokine-associated genes are determinants of mortality in prevalent HD patients.

## Methods

### Patients

HD patients living in the Greater Poland District, Poland, were enrolled into the prospective, observational study in January, 2009. Known status in respect to HBV susceptibility or infection was an inclusion criterion, because the ability to produce antibodies to HBV surface antigen (anti-HBs) was one of parameters investigated as a predictor of survival [10]. An exclusion criterion was renal transplantation prior to enrolment. Patients were in stable clinical condition for at least one month prior to enrolment.

Characteristics of enrolled patients (*n* = 532) are shown in our previous publication [10]. In brief, the study included 297 men and 235 women in the age of 61.2 (14.6–89.3) years. Dialysis vintage prior to the study onset was 2.2 (0.0–24.7) years. The main cause of end-stage renal disease was diabetic nephropathy (*n* = 137, 25.8% of all). Patients were dialyzed using low-flux HD (*n* = 277, 52.0% of all), high-flux HD (*n* = 217, 40.8% of all), and on-line hemodiafiltration (*n* = 38, 7.1% of all).

Patients were followed from January 30, 2009 to January 30, 2016. The study was planned initially to January 30, 2015, however, we decided to prolong the patient monitoring for the additional year to evaluate a possible association of genetic factors with survival [11]. During the 7-year prospective study, 317 (59.6% of all) patients died, 66 (12.4% of all) underwent renal transplantation, and 7 (1.3% of all) moved to non-collaborating dialysis centers. Main causes of death included cardiovascular (*n* = 203, 64.0% of all deaths), infection (*n* = 39, 12.3% of all deaths), and neoplasm (*n* = 30, 9.5% of all deaths) reasons.

### Genotyping

High-resolution melting curve (HRM) analysis was used for interferon lambda 3 gene (*IFNL3*), interleukin 12A gene (*IL12A*), interleukin 13 gene (*IL13*), and interleukin 4 receptor gene (*IL4R*) genotyping. Chemokine (C-C motif) ligand 2 gene (*CCL2*), interleukin 12B gene (*IL12B*), and interleukin 18 gene (*IL18*) were genotyped using the polymerase chain reaction–restriction fragment length polymorphism (PCR-RFLP) analysis. Genotyping was performed as previously described [9, 11–13].

In brief, genomic DNA for genotype analyses was isolated from peripheral blood lymphocytes by a salt-out extraction procedure. The characteristics of analyzed polymorphisms are described in Additional file 1: Table S1. Primer sequences and conditions for PCR-RFLP and HRM analyses are presented in Additional file 1: Table S2. Approximately 10% of the randomly chosen samples were re-genotyped. Samples that failed the genotyping were excluded from further statistical analyses.

Genotyping of tested single nucleotide polymorphisms (SNPs) was performed in groups of 418–524 patients (Additional file 1: Table S3).

### Statistical methods

The results are presented as numbers and percentages for categorical variables. Medians and ranges for continuous variables are shown as data sets were non-normally distributed by the Shapiro–Wilk test in the majority of subgroups.

The Hardy–Weinberg equilibrium was analyzed to compare the observed genotype frequencies to the expected ones using the Chi-square test (*P* > 0.05 with df = 1 for equilibrium).

Survival analyses were conducted using the Kaplan-Meier method with the log rank test or with calculation of multiple *P* value when more than two groups were compared. The Cox proportional hazard model was applied to show whether and to which extend the effect of a unit increase in a covariate was multiplicative with respect to the hazard rate of death.

Cox proportional hazard model was also applied in multivariate analyses assessing the contribution of demographics and clinical measures to mortality.

An effect size for the power equal to 0.8 was estimated for Mann-Whitney test and Chi-square test. The power of any test was calculated for statistically significant outcomes.

Abovementioned statistical analyses were performed using Graph-Pad InStat 3.10, 32 bit for Windows (GraphPad Software, Inc., San Diego, California, United States), Statistica version 12 (Stat Soft, Inc., Tulsa, Oklahoma, United States), and G*Power 3.1.9.2 (Franz Faul, Universitat Kiel, Germany).

Haplotype frequencies were estimated using the Haploview 4.2 software (http://www.broad.mit.edu/mpg/haploview/). Statistical significance was assessed using the 1000-fold permutation test.

Epistatic interactions were analyzed using the logistic regression and epistasis option in the PLINK software (http://pngu.mgh.harvard.edu/purcell/plink/).

A *P* value of less than 0.05 was considered significant.

## Results

At the beginning of the study, all tested polymorphisms were in concordance with HWE (Additional file 1: Table S3).

### Univariate survival analyses

In univariate survival analyses, longer survival was attributed to chronic glomerulonephritis and polycystic kidney disease as causes of end-stage renal disease (ESRD), and the ability to develop anti-HBs in response to HBV vaccination or infection. Shorter survival was demonstrated in patients with older age at the beginning of the study, coronary artery disease (CAD), diabetic nephropathy, and lower serum parathyroid hormone (PTH) concentrations. The details of abovementioned analyses are shown in our earlier paper [11].

*IFNL3* rs8099917 was associated with all-cause mortality in recessive model of inheritance, *IL12A* rs568408 - in dominant model (Table 1). Both these associations were relatively weak (*P* = 0.044 for *IFNL3* rs8099917 and *P* = 0.029 for *IL12A* rs568408).

**Table 1:** Differences in all-cause mortality by T helper cell cytokine genes in hemodialysis patients

Tested polymorphism	N	Major homozytes vs. heterozygotes vs. minor homozygotes^a^	Dominant model of inheritance^b^	Recessive model of inheritance^b^	Additive model of inheritance^b^
*CCL2* rs1024611	261	AA vs. AG vs. GG *P* = 0.848	GG + AG vs. AA *P* = 0.363	GG vs. AG + AA *P* = 0.777	GG vs. AA *P* = 0.601
*IFNL3* rs8099917	265	TT vs. GT vs. GG *P* = 0.139	GG + GT vs. TT *P* = 0.688	GG vs. GT + TT *P* = 0.044^c^	GG vs. TT *P* = 0.052
*IFNL3* rs12979860	266	CC vs. CT vs. TT *P* = 0.394	TT + CT vs. CC *P* = 0.896	TT vs. CT + CC *P* = 0.490	TT vs. CC *P* = 0.640
*IL4R* rs1805015	294	TT vs. CT vs. CC *P* = 0.829	CC + CT vs. TT *P* = 0.703	CC vs. CT + TT *P* = 0.352	CC vs. TT *P* = 0.348
*IL12A* rs568408	297	GG vs. AG vs. AA *P* = 0.034	AA + AG vs. GG *P* = 0.029^d^	AA vs. AG + GG *P* = 0.816	AA vs. GG *P* = 0.958
*IL12B* rs3212227	297	AA vs. AC vs. CC *P* = 0.274	CC + AC vs. AA *P* = 0.163	CC vs. AC + AA *P* = 0.104	CC vs. AA *P* = 0.087
*IL13* rs20541	293	CC vs. CT vs. TT *P* = 0.412	TT + CT vs. CC *P* = 0.189	TT vs. CT + CC *P* = 0.981	TT vs. CC *P* = 0.803
*IL18* rs360719	316	TT vs. CT vs. CC *P* = 0.651	CC + CT vs. TT *P* = 0.260	CC vs. CT + TT *P* = 0.648	CC vs. TT *P* = 0.484

*Abbreviations*: *CCL2* chemokine (C-C motif) ligand 2 gene, *IFNL3* interferon lambda 3 gene, *IL* interleukin gene, *IL4R* interleukin 4 receptor gene, *N* number of deaths among tested patients

^a^Multiple-sample test P

^b^Log rank test P

^c^The test power = 0.98

^d^The test power = 0.66

**Fig. 1:**
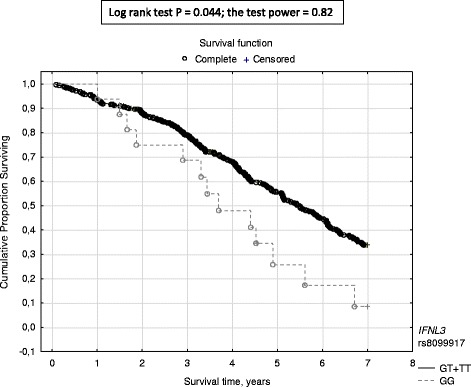
The probability of survival in hemodialysis patients in respect to *IFNL3* rs8099917 polymorphic variant

Minor homozygotes (the genotype GG) in *IFNL3* rs8099917 showed shorter survival during the study (3.6, 1.0–7.0 years vs 4.7, 0.1–7.0 years, *P* = 0.009) than the major allele (T) bearers, although their renal replacement therapy (RRT) vintage prior to the onset of the study was also shorter (1.4, 0.0–6.8 years vs 2.3, 0.0–22.2 years, *P* = 0.010) (Additional file 1: Table S4). The rs8099917 GG patients demonstrated higher risk of death (HR 1.944, 95% CI 1.112–3.401) than the remaining patients (GT + TT), (Fig. 1).

**Fig. 2:**
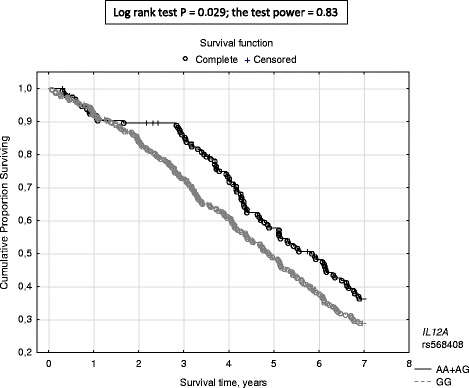
The probability of survival in hemodialysis patients in respect to *IL12A* rs568408 polymorphic variant

Major homozygosity (the genotype GG) in *IL12A* rs568408 was associated with higher mortality (HR 1.313, 95% CI 1.20–1.691) than that shown in bearers of the minor allele (AA + AG) (Fig. 2). There were 9.7% less responders to HBV vaccination in HBV non-infected patients showing the genotype GG compared with patients harboring the minor allele in rs568408 (Additional file 1: Table S5).

There were no significant associations between cardiovascular (Additional file 1: Table S6), infection-related (Additional file 1: Table S7) or neoplasm-related (Additional file 1: Table S8) mortalities and tested polymorphisms.

### Multivariate analyses of survival

Basic variables that yielded significance in univariate analyses of survival (age at the beginning of the study, diabetic nephropathy, polycystic kidney disease and chronic glomerulonephritis as causes of ESRD, CAD, the ability to develop anti-HBs in response to HBV vaccination or infection, and serum PTH concentration) as well as RRT vintage prior to the study onset were used in multivariate analysis. CAD (HR 1.753, 95% CI 1.383–2.221, *P* = 0.000003), age at the beginning of the study (HR 1.019, 95% CI 1.009–1.029, *P* = 0.0001), RRT vintage prior to the study onset (HR 1.058, 95% CI 1.021–1.097, *P* = 0.002), and the ability to develop antibodies to HBV surface antigen in response to HBV vaccination or infection (HR 0.672, 95% CI 0.493–0.915, *P* = 0.012) remained as independent predictors of 7-year survival of prevalent HD patients.

When both SNPs showing significance in univariate analyses (*IFNL3* rs8099917 and *IL12A* rs568408) were used in multivariate analyses including all 8 basic variables or only 4 variables that remained significant, the tested SNPs did not yield significance. Moreover, the ability to develop anti-HBs lost its significance as an independent predictor of survival. When *IFNL3* rs8099917 and *IL12A* rs568408 were used separately in multivariate analyses, only *IFNL3* rs8099917 abolished significance of anti-HBs.

### Haplotype frequencies and epistatic interactions

Haplotype analysis of *IFNL3* polymorphic variants did not reveal significance (Additional file 1: Table S9) as well as there were no significant gene-gene interactions between tested polymorphisms (Additional file 1: Table S10) in the 7-year survivors and non-survivors.

## Discussion

In this study, we were not able to show independent predictors of all-cause mortality of HD patients among tested polymorphic variants of Th cell cytokine related genes. Similarly like in our retrospective study [9], the GG genotype in *IFNL3* rs8099917 was associated with worse survival probability in HD patients, however, only in univariate analysis. Additionally, the GG genotype in *IL12A* rs568408 was added as possible having relation with all-cause mortality.

IL-12 is a heterodimeric pro-inflammatory cytokine that stimulates the differentiation of Th1 cells [14]. It is formed by a 35-kDa light chain (known as IL-12A or p35) and a 40-kDa heavy chain (known as IL-12A or p40) [14]. The subunits IL-12A and IL12-B are encoded by *IL12A* and *IL12B*, respectively, which are located on separate chromosomes (3p12-q13.2 and 5q31-33) [15]. IL-12 induces T-cell recruitment into the atherosclerotic plaque [16]. In the study by Mishra et al. [17], IL-12 correlated with endothelial dysfunction, insulin resistance and pro-inflammatory markers in type 2 diabetes patients. The minor allele in *IL12A* rs568408 has been associated with numerous neoplasms such as hepatocellular carcinoma [18], cervical cancer [19], colorectal cancer [20] or osteosarcoma [21]. *IL12A* rs568408 seems to be associated with autoimmune disorders: the A allele in *IL12A* rs568408 was found to be significantly higher in patients with Graves’ disease than in controls [22]. According to Jiang et al. [23], patients with oral lichen planus were more likely to have the *IL12A* rs568408 A allele and this allele was also associated with the severity of oral lichen planus. *IL12A* rs568404 might also contribute to the risk of asthma [24]. Abovementioned pathologic findings related to *IL12A* rs568408 and IL-12A were not documented in HD patients yet. However, the GG genotype in *IL12A* rs568408 was shown as associated with the impaired anti-HBs development in our previous studies on Caucasian HD patients [12]. Additionally, there was a lower peak of anti-HBs titers in the GG genotype HD patients compared with those with one or two minor alleles [25]. In the study by Pan et al. [26], the T allele in *IL12A* rs2243115 contributed to the risk of low response to HBV vaccination in a Chinese Han population. In the current study group, anti-HBs generation was documented as a significant independent predictor of 6-year [10] and 7-year survival. The *IL12A* rs568408 GG genotype possessors revealed about 10% lower responders to HBV vaccination than the remaining patients, and they showed lower survival probability.

IFN-λ3, a cytokine encoded by *IFNL3*, belongs to the family of type III IFNs and plays a role in the immune response through the activation of the Th1 pathway [27]. IFN-λ3 was also found as up-regulator of indoleamine 2,3-dioxygenase (IDO) expression [28]. Increased IDO activity seems to skew helper T-cell polarization toward a Th2 phenotype [29]. IFN-λ3 has been shown to be a potent antiviral molecule [30]. IFN-λ3, similarly to other type III IFNs, is activated during bacterial infections [31]. *IFNL3* is located on chromosomal region mapped to 19q13 [32]. rs12979860 and rs8099917 *IFNL3* SNPs were involved in the production of IFN-λ3 [33]. *IFNL3* rs8099917 polymorphisms seem to be associated with IFN-λ3 plasma levels also in HD subjects [34]. Major alleles of rs12979860 and rs8099917 have been found to influence the response of HCV-infected patients to pegylated-IFN/ribavirin therapy [35]. *IFNL3* polymorphisms were associated with spontaneous resolution of HCV infection in the general population [33] and in HD subjects [13] as well as IFN-related HBsAg seroclearance in chronic hepatitis B patients [36]. *IFNL3* rs12979860 and rs8099917 polymorphisms have been found to affect the risk of hepatitis virus-related hepatocellular carcinoma [37]. Major alleles of *IFNL3* are associated with less pronounced disturbances of lipid metabolism and less frequent steatosis and insulin resistance in chronic hepatitis C patients [38].

The GG genotype in *IFNL3* rs8099917 was associated in the current study with worse survival of HD patients. The G allele in *IFNL3* rs8099917 was attributed to lower circulating IFN-λ3 than that shown in the TT genotype subjects [34]. Circulating IFN-λ3 strongly correlates with anti-HBs production after HBV vaccination and infection in this group of patients [34]. However, a direct association between *IFNL3* polymorphic variants and anti-HBs generation was not demonstrated [13], although both are related to survival of HD patients. This lack of such association may occur due to possibility that the association between plasma IFN-λ3 and anti-HBs titers is also indirect. Our suggestion is that a link between them is provided by IDO which is involved in anti-HBs production [39]. At every step of anti-HBs production, additional factors, example related to uremic milieu and dialysis procedure [40], may disturb existing associations.

## Conclusions

Polymorphisms of Th cell cytokine-associated genes (*IFNL3* rs8099917, *IL12A* rs568408) are associated with survival on HD, however, they are not independent predictors of mortality in HD patients.

The independent association of anti-HBs with survival may be at least partially explained by polymorphisms in Th cell cytokine-associated genes.

## Additional file

Additional file 1: Supplementary material for polymorphisms of T helper cell cytokine-associated genes in respect to survival of hemodialysis patients – a prospective observational study. Description of data: Supplementary material contains characteristics, conditions for the identification and genotype distribution of the analyzed polymorphisms; characteristics of patients bearing different polymorphic variants of tested genes; cardiovascular, infection-related, and neoplasm-related mortality evaluated by the Kaplan-Meier analysis in respect of T helper cell cytokine genes; and haplotype and epistatic gene-gene interaction analyses. (DOCX 56 kb)

## Abbreviations

ALP: Alkaline phosphatase
ALT: Alanine aminotransferase
Anti-HBc: Antibodies to core antigen of hepatitis B virus
Anti-HBs: Antibodies to surface antigen of hepatitis B virus
AST: Aspartate aminotransferase
CAD: Coronary artery disease
*CCL2*: Chemokine (C-C motif) ligand 2 gene
ESRD: End-stage renal disease
GGT: Gamma-glutamyl transferase
HBsAg: Surface antigen of hepatitis B virus
HBV: Hepatitis B virus
HCV: Hepatitis C virus
HD: Hemodialysis
HDF: Hemodiafiltration
HF-HD: High flux hemodialysis
HRM analysis: High Resolution Melt analysis
IDO: Indoleamine 2,3-dioxygenase
IFN: Interferon
*IFNL3*: Interferon lambda 3 gene
*IL*: Interleukin gene
*IL4R*: Interleukin 4 receptor gene
LF-HD: Low flux hemodialysis
N: Number of patients
PD: Peritoneal dialysis
PTH: Parathyroid hormone
RFLP analysis: Restriction Fragment Length Polymorphism analysis
RNA: Ribonucleic acid
RRT: Renal replacement therapy
SNP: Single nucleotide polymorphism
Th: T helper
