# Supplementary material for: Polymorphisms of T helper cell cytokine-associated genes and survival of hemodialysis patients – a prospective study
Source: BMC Nephrol. 2017 May 19;18:165. doi: 10.1186/s12882-017-0582-x (PMC5437603; doi:10.1186/s12882-017-0582-x)
Supplement: Supplementary file 1 — Supplementary material for polymorphisms of T helper cell cytokine-associated genes in respect to survival of hemodialysis patients – a prospective observational study. Description of data: Supplementary material contains characteristics, conditions for the identification and genotype distribution of the analyzed polymorphisms; characteristics of patients bearing different polymorphic variants of tested genes; cardiovascular, infection-related, and neoplasm-related mortality evaluated by the Kaplan-Meier analysis in respect of T helper cell cytokine genes; and haplotype and epistatic gene-gene interaction analyses. (DOCX 56 kb) [file 12882_2017_582_MOESM1_ESM.docx]

Additional file 1 for polymorphisms of T helper cell cytokine-associated genes in respect to survival of hemodialysis patients – a prospective observational study

Alicja E. Grzegorzewska, Chair and Department of Nephrology, Transplantology and Internal Diseases, Poznan University of Medical Sciences, Poznań, Poland; e-mail: alicja_grzegorzewska@yahoo.com

Monika K. Świderska, Student Nephrology Research Group, Chair and Department of Nephrology, Transplantology and Internal Diseases, Poznan University of Medical Sciences, 5 Poznań, Poland; e-mail: monika.swi@gmail.com

Adrianna Mostowska, Chair and Department of Biochemistry and Molecular Biology, Poznan University of Medical Sciences, Poznań, Poland; e-mail: amostowska@wp.pl

Wojciech Warchoł, Chair and Department of Biophysics, Poznan University of Medical Sciences, Poznań, Poland; e-mail: wwarchol@ump.edu.pl

Paweł P. Jagodziński, Chair and Department of Biochemistry and Molecular Biology, Poznan University of Medical Sciences, Poznań, Poland; e-mail: pjagodzi@ump.edu.pl

Table S1. Characteristics of the analyzed polymorphisms

| Gene symbol | rs no. | Location^a^ | Alleles^b^ | SNP function^c^ | MAF^d^ |
| --- | --- | --- | --- | --- | --- |
| *CCL2* | rs1024611 | chr17:32579788 | C / T | N/A | 0.316 |
| *IFNL3* | rs8099917 | chr19:39743165 | G / T | N/A | 0.168 |
| *IFNL3* | rs12979860 | chr19:39738787 | C / T | Intron | 0.309 |
| *IL4R* | rs1805015 | chr16:27374180 | C / T | Missense (p.Ser503Pro) | 0.152 |
| *IL12A* | rs568408 | chr3:159713467 | A / G | UTR-3 | 0.117 |
| *IL12B* | rs3212227 | chr5:158742950 | A / C | UTR-3 | 0.223 |
| *IL13* | rs20541 | chr5:131995964 | C / T | Missense (p.Gln144Arg) | 0.207 |
| *IL18* | rs360719 | chr11:112036149 | A / G | NearGene-5 | 0.278 |

^a^  - NCBI build 37 / hg19

^b^ - Underline denotes the minor allele

^c^ - According to the Single Nucleotide Polymorphism database (dbSNP)

^d^ - MAF, minor allele frequency calculated from 1000 Genomes project for EUR samples

Abbreviations: *CCL2* - chemokine (C-C motif) ligand 2 gene, *IFNL3* - interferon lambda 3 gene, *IL* - interleukin gene, *IL4R* - interleukin 4 receptor gene, MAF, minor allele frequency calculated from 1000 Genomes project for EUR samples, SNP – single nucleotide polymorphism, UTR – untranslated region

Table S2. HRM and RFLP conditions for the identification of genotyped polymorphisms

|  |  |  |  |  |  | HRM analysis | RFLP analysis | |
| --- | --- | --- | --- | --- | --- | --- | --- | --- |
| Gene symbol | rs no. | Alleles | Primers for PCR amplification  (5’ – 3’) | Annealing temp. (°C) | PCR product length (bp) | Melting temp. range (°C) | Restriction enzyme | Restriction fragment length (bp) |
| *CCL2* | rs1024611 | A/G | F: CTTTCCCTTGTGTGTCCCC | 66.3 | 940 |  | PvuII | A = 940 |
|  |  |  | R: TTACTCCTTTTCTCCCCAACC |  |  |  |  | G = 650 + 290 |
| *IFNL3* | rs8099917 | T/G | F: TTTGTCACTGTTCCTCCTTTTG | 61.0 | 98 | 76 - 86 |  |  |
|  |  |  | R: AAGACATAAAAAGCCAGCTACCA |  |  |  |  |  |
|  | rs12979860 | C/T | F: CGTGCCTGTCGTGTACTGAA | 61.0 | 148 | 86 - 96 |  |  |
|  |  |  | R: AGGCTCAGGGTCAATCACAG |  |  |  |  |  |
| *IL4R* | rs1805015 | T/C | F: AACCCTGCTTACCGCAGCTT | 60.6 | 107 | 83 - 93 |  |  |
|  |  |  | R: TCGGGTTCTACTTCCTCCAGGT |  |  |  |  |  |
| *IL12A* | rs568408 | G/A | F: ATGAGGAAACTTTGATAGGATG | 54.0 | 156 | 70 - 85 |  |  |
|  |  |  | R: TTCCCTTCTTAGCAATTCATTC |  |  |  |  |  |
| *IL12B* | rs3212227 | A/C | F: TTAAAGACACAACGGAATAGAC | 62.6 | 557 |  | TaqI | A = 557 |
|  |  |  | R: TGCTTTATCAACACCATCTCC |  |  |  |  | C = 455 + 102 |
| *IL13* | rs20541 | C/T | F: CCAGTTTGTAAAGGACCTGCT | 60.6 | 97 | 75 - 85 |  |  |
|  |  |  | R: CCTGTCTCTGCAAATAATGATG |  |  |  |  |  |
| *IL18* | rs360719 | T/C | F: CAACAGTGATTACAAAGGAAGT | 62.6 | 474 |  | NlaIII | T = 295 + 179 |
|  |  |  | R: TAAATGGGTAGGAATAAGTGAGA |  |  |  |  | C = 474 |

Abbreviations: *CCL2* - chemokine (C-C motif) ligand 2 gene, HRM analysis - High Resolution Melt analysis, *IFNL3* - interferon lambda 3 gene, *IL* - interleukin gene, *IL4R* - interleukin 4 receptor gene, RFLP analysis - Restriction Fragment Length Polymorphism analysis

Table S3. Genotype distribution of tested polymorphisms

| Tested SNP | N | Major homozygote  n, frequency | Heterozygote  n, frequency | Minor homozygote  n, frequency | *P* value for HWE  df = 1 |
| --- | --- | --- | --- | --- | --- |
| *CCL2* rs1024611 | 418 | 202 (0.48) | 184 (0.44) | 32 (0.08) | 0.262 |
| *IFNL3* rs8099917 | 441 | 282 (0.64) | 143 (0.32) | 16 (0.04) | 0.684 |
| *IFNL3* rs12979860 | 444 | 185 (0.42) | 198 (0.44) | 61 (0.14) | 0.491 |
| *IL4R* rs1805015 | 484 | 335 (0.70) | 128 (0.26) | 21 (0.04) | 0.057 |
| *IL12A* rs568408 | 485 | 338 (0.70) | 133 (0.27) | 14 (0.03) | 0.834 |
| *IL12B* rs3212227 | 484 | 292 (0.60) | 174 (0.36) | 18 (0.04) | 0.201 |
| *IL13* rs20541 | 481 | 265 (0.55) | 190 (0.40) | 26 (0.05) | 0.282 |
| *IL18* rs360719 | 524 | 260 (0.50) | 230 (0.44) | 34 (0.06) | 0.072 |

Abbreviations: *CCL2* - chemokine (C-C motif) ligand 2 gene, *IFNL3* - interferon lambda 3 gene, *IL* - interleukin gene, *IL4R* - interleukin 4 receptor gene

Table S4. Characteristics of minor allele homozygotes and patients bearing the major allele in *IFNL3* rs8099917

| Parameter | *IFNL3* rs8099917 GG  N = 16 | *IFNL3* rs8099917  GT + TT  N = 425 | P value^a^ |
| --- | --- | --- | --- |
| Demographic data |  |  |  |
| Male gender, n, % of all | 10 (62.5) | 240 (56.5) | 0.825 |
| Age at the beginning of the study, years | 58.0 (29.2 - 83) | 61.4 (14.6 - 89.3) | 0.827 |
| Age at the beginning of RRT, years | 56.5 (29.2 - 82.5) | 58.6 (11.1 - 86.6) | 0.867 |
| RRT vintage prior to the study onset, years | 1.4 (0.0 - 6.8) | 2.3 (0.0 - 22.2) | 0.010 |
| Total RRT vintage, years | 5.3 (1.6 - 13.8) | 7.5 (0.5 - 28.3) | 0.007 |
| HD vintage on the prospective study, years | 3.6 (1.0 – 7.0) | 4.7 (0.1 – 7.0) | 0.009 |
| Time since RRT onset to renal transplantation, years | 4.9 (3.0 - 6.7) | 4.5 (0.6 - 20.7) | 0.752 |
| Cause of ESRD |  |  |  |
| Diabetic nephropathy, n, % of all | 4 (25.0) | 112 (26.4) | 1.000 |
| Chronic glomerulonephritis, n, % of all | 4 (25.0) | 68 (16.0) | 0.541 |
| Hypertensive nephropathy, n, % of all | 3 (18.8) | 73 (17.2) | 1.000 |
| Chronic tubulointerstitial nephritis, n, % of all | 1 (6.2) | 49 (11.5) | 0.801 |
| Polycystic kidney disease, n, % of all | 0 (0.0) | 26 (6.1) | 0.632 |
| Clinical data |  |  |  |
| Coronary artery disease, n, % of all | 10 (62.5) | 171 (40.2) | 0.135 |
| Myocardial infarction, n, % of all | 7 (43.8) | 96 (22.6) | 0.100 |
| Responders to HBV vaccination among HBV non-infected patients, n/N, % of N | 10/14 (71.4) | 287/322 (89.1) | 0.110 |
| Maximum anti-HBs titer among vaccine responders | 169.5 (22.2 - 2000) | 396.4 (10.0 - 5342) | 0.885 |
| History of HBV infection (anti-HBc positivity), n, % of all | 2 (12.5) | 103 (24.2) | 0.428 |
| History of HCV infection (anti-HCV positivity), n, % of all | 3 (18.8) | 48 (11.3) | 0.605 |
| HBsAg positivity, n, % of all | 0 (0.0) | 14 (3.3) | 0.991 |
| HCV RNA positivity, n, % of all | 3 (18.8) | 30 (7.1) | 0.207 |
| Renal transplantation, n, % of all | 2 (12.5) | 50 (11.8) | 1.000 |
| Cinacalcet treatment/ Parathyroidectomy, n, % of all | 2 (12.5) | 116 (27.3) | 0.306 |
| Dyslipidemia, n, % of all | 9 (56.2) | 191 (44.9) | 0.525 |
| Type of RRT |  |  |  |
| LF-HD, n, % of all | 10 (62.5.3) | 225 (52.9) | 0.619 |
| HF-HD, n, % of all | 6 (37.5) | 164 (38.6) | 1.000 |
| HDF, n, % of all | 0 (0.0) | 36 (8.5) | 0.453 |
| HF-HD/HDF, n, % of all | 6 (37.5) | 200 (47.1) | 0.619 |
| PD as the first modality of RRT, n, % of all | 1 (6.2) | 15 (3.5) | 1.000 |
| Laboratory data |  |  |  |
| ALT, IU/L | 11.5 (2.0 – 72.0) | 13 (2 - 131) | 0.349 |
| AST, IU/L | 16.5 (4.0 – 60.0) | 15 (3 - 177) | 0.197 |
| GGT, IU/L | 36 (4 - 308) | 26 (1 - 682) | 0.335 |
| ALP, U/L | 94 (58.5 - 123.5) | 97.8 (38.3 - 1684) | 0.317 |
| PTH, pg/mL | 450.5 (115 - 1081.5) | 434 (16.8 - 3757) | 0.884 |
| Ca, mg/dL | 8.9 (7.2 - 9.7) | 8.9 (5.4 - 11.7) | 0.600 |
| P, mg/dL | 5.2 (2.2 - 6.9) | 5.1 (2.2 - 11.3) | 0.743 |
| Causes of death |  |  |  |
| All, n, % of all | 13 (81.2) | 252 (59.3) | 0.133 |
| Cardiovascular, n, % of all | 8 (50.0) | 164 (38.6) | 0.511 |
| Cardiac, n, % of all | 2 (12.5) | 109 (25.6) | 0.370 |
| Sepsis/infection, n, % of all | 2 (12.5) | 31 (7.3) | 0.770 |
| Neoplasms, n, % of all | 1 (6.2) | 20 (4.7) | 1.000 |
| Rare/unknown, n, % of all | 2 (12.5) | 37 (8.7) | 0.939 |

a – Mann-Whitney U test was used for comparison of continuous variables. Chi square test with Yates correction was applied for comparison of dichotomous variables.

Abbreviations:

Abbreviations: ALP - alkaline phosphatase, ALT – alanine aminotransferase, Anti-HBc - antibodies to core antigen of hepatitis B virus, Anti-HBs - antibodies to surface antigen of hepatitis B virus, AST - aspartate aminotransferase, ESRD – end-stage renal disease, GGT - gamma-glutamyl transferase, HBsAg - surface antigen of hepatitis B virus, HBV - hepatitis B virus, HCV - hepatitis C virus, HD – hemodialysis, HDF - hemodiafiltration , HF-HD - high flux hemodialysis, LF-HD - low flux hemodialysis, N - number of patients, PD - peritoneal dialysis, PTH - parathyroid hormone, RNA - ribonucleic acid, RRT - renal replacement therapy

Table S5. Characteristics of major allele homozygotes and minor allele bearers in *IL12A* rs568408

| Parameter | *IL12A* rs568408  GG  N = 338 | *IL12A* rs568408  AA+AG  N = 147 | P value^a^ |
| --- | --- | --- | --- |
| Demographic data |  |  |  |
| Male gender, n, % of all | 189 (55.9) | 85 (57.8) | 0.772 |
| Age at the beginning of the study, years | 61.9 (14.6 - 89.3) | 60.4 (18.1 - 86.7) | 0.913 |
| Age at the beginning of RRT, years | 58.5 (11.1 - 86.5) | 58.6 (17.5 - 86.6) | 0.810 |
| RRT vintage prior to the study onset, years | 2.2 (0 - 24.7) | 2.3 (0 - 22.2) | 0.602 |
| Total RRT vintage, years | 7.1 (0.5 - 24.8) | 7.6 (0.5 - 28.3) | 0.512 |
| HD vintage on the prospective study, years | 4.1 (0.1 – 7.0) | 4.9 (0.3 – 7.0) | 0.062 |
| Time since RRT onset to renal transplantation, years | 4.4 (0.7 - 19.9) | 3.9 (0.6 - 15.6) | 0.514 |
| Cause of ESRD |  |  |  |
| Diabetic nephropathy, n, % of all | 95 (28.1) | 33 (22.4) | 0.235 |
| Chronic glomerulonephritis, n, % of all | 57 (16.9) | 26 (17.7) | 0.928 |
| Hypertensive nephropathy, n, % of all | 48 (14.2) | 33 (22.4) | 0.035 |
| Chronic tubulointerstitial nephritis, n, % of all | 45 (13.3) | 10 (6.8) | 0.055 |
| Polycystic kidney disease, n, % of all | 19 (5.6) | 11 (7.5) | 0.564 |
| Clinical data |  |  |  |
| Coronary artery disease, n, % of all | 139 (41.1) | 63 (42.9) | 0.858 |
| Myocardial infarction, n, % of all | 82 (24.3) | 32 (21.8) | 0.598 |
| Responders to HBV vaccination among HBV non-infected patients, n/N, % of N | 220/260 (84.6) | 99/105 (94.3) | 0.019 |
| Maximum anti-HBs titer among vaccine responders | 329.8 (13 - 4105) | 302.3 (12 - 1000) | 0.267 |
| Anti-HBs positivity |  |  |  |
| History of HBV infection (anti-HBc positivity), n, % of all | 78 (23.1) | 42 (28.6) | 0.255 |
| History of HCV infection (anti-HCV positivity), n, % of all | 41 (12.1) | 13 (8.8) | 0.368 |
| HBsAg positivity, n, % of all | 7 (2.1) | 7 (4.8) | 0.183 |
| HCV RNA positivity, n, % of all | 25 (7.4) | 9 (6.1) | 0.755 |
| Renal transplantation, n, % of all | 45 (13.3) | 17 (11.6) | 0.702 |
| Cinacalcet treatment, n, % of all | 62 (18.3) | 40 (27.2) | 0.044 |
| Parathyroidectomy, n, % of all | 17 (5.0) | 12 (8.2) | 0.729 |
| Dyslipidemia, n, % of all | 150 (44.4) | 69 (46.9) | 0.841 |
| Type of RRT |  |  |  |
| LF-HD, n, % of all | 176 (52.1) | 83 (56.5) | 0.428 |
| HF-HD, n, % of all | 134 (39.6) | 56 (38.1) | 0.826 |
| HDF, n, % of all | 28 (8.3) | 8 (5.4) | 0.363 |
| HF-HD/HDF, n, % of all | 162 (47.9) | 64 (43.5) | 0.428 |
| PD as the first modality of RRT, n, % of all | 11 (3.3) | 6 (4.1) | 0.880 |
| Laboratory data |  |  |  |
| ALT, IU/L | 13 (2 - 126) | 13 (2 - 131) | 0.339 |
| AST, IU/L | 15 (3 - 97) | 14 (5 - 177) | 0.228 |
| GGT, IU/L | 27 (1 - 682) | 23.5 (5 - 323) | 0.144 |
| ALP, U/L | 97.9 (42.5 - 1684) | 98.3 (38.3 - 1353.3) | 0.245 |
| PTH, pg/mL | 414.5 (12.7 - 3740.7) | 481.4 (52 - 3757) | 0.930 |
| PTH < 150 pg/mL, n, % of all | 52 (15.4) | 18 (12.2) | 0.445 |
| Ca, mg/dL | 8.9 (5.4 - 11.7) | 8.9 (7 - 10.6) | 0.859 |
| P, mg/dL | 5.1 (2.4 - 10.5) | 5 (2.2 - 11.3) | 0.788 |
| Causes of death |  |  |  |
| All, n, % of all | 213 (63.0) | 84 (57.1) | 0.263 |
| Cardiovascular, n, % of all | 137 (40.5) | 53 (36.1) | 0.408 |
| Cardiac, n, % of all | 98 (29.0) | 32 (21.8) | 0.124 |
| Sepsis/infection, n, % of all | 22 (6.5) | 15 (10.2) | 0.221 |
| Neoplasms, n, % of all | 20 (5.9) | 7 (4.8) | 0.768 |
| Rare/unknown, n, % of all | 34 (10.1) | 9 (6.1) | 0.219 |

a – Mann-Whitney U test was used for comparison of continuous variables. Chi square test with Yates correction was applied for comparison of dichotomous variables.

Abbreviations:

Abbreviations: ALP - alkaline phosphatase, ALT – alanine aminotransferase, Anti-HBc - antibodies to core antigen of hepatitis B virus, Anti-HBs - antibodies to surface antigen of hepatitis B virus, AST - aspartate aminotransferase, ESRD – end-stage renal disease, GGT - gamma-glutamyl transferase, HBsAg - surface antigen of hepatitis B virus, HBV - hepatitis B virus, HCV - hepatitis C virus, HD – hemodialysis, HDF - hemodiafiltration , HF-HD - high flux hemodialysis, LF-HD - low flux hemodialysis, N - number of patients, PD - peritoneal dialysis, PTH - parathyroid hormone, RNA - ribonucleic acid, RRT - renal replacement therapy

Table S6. Statistical significance of differences in cardiovascular mortality evaluated by the Kaplan-Meier analysis for T helper cell cytokine genes in hemodialysis patients undergoing the 7-year prospective study

| Tested polymorphism | N | Major homozytes vs. heterozygotes vs. minor homozygotes^a^ | Dominant model of inheritance^b^ | Recessive model of inheritance^b^ | Additive model of inheritance^b^ |
| --- | --- | --- | --- | --- | --- |
| *CCL2* rs1024611 | 171 | AA vs. AG vs. GG  P = 0.283 | GG + AG vs. AA  P = 0.336 | GG vs. AG + AA  P = 0.236 | GG vs. AA  P = 0.177 |
| *IFNL3* rs8099917 | 172 | TT vs. GT vs. GG  P = 0.135 | GG + GT vs. TT  P = 0.061 | GG vs. GT + TT  P = 0.759 | GG vs. TT  P = 0.899 |
| *IFNL3* rs12979860 | 171 | CC vs. CT vs. TT  P = 0.290 | TT + CT vs. CC  P = 0.324 | TT vs. CT + CC P = 0.798 | TT vs. CC  P = 0.765 |
| *IL4R* rs1805015 | 191 | TT vs. CT vs. CC  P = 0.816 | CC + CT vs. TT  P = 0.620 | CC vs. CT + TT  P = 0.436 | CC vs. TT  P = 0.385 |
| *IL12A* rs568408 | 190 | GG vs. AG vs. AA P = 0.404 | AA + AG vs. GG P = 0.133 | AA vs. AG + GG  P = 0.783 | AA vs. GG  P = 0.905 |
| *IL12B* rs3212227 | 189 | AA vs. AC vs. CC  P = 0.664 | CC + AC vs. AA  P = 0.489 | CC vs. AC + AA  P = 0.162 | CC vs. AA  P = 0.197 |
| *IL13* rs20541 | 190 | CC vs. CT vs. TT  P = 0.929 | TT + CT vs. CC  P = 0.945 | TT vs. CT + CC P = 0.378 | TT vs. CC  P = 0.326 |
| *IL18* rs360719 | 202 | TT vs. CT vs. CC  P = 0.930 | CC + CT vs. TT  P = 0.344 | CC vs. CT + TT  P = 0.702 | CC vs. TT  P = 0.653 |

Abbreviations: *CCL2* - chemokine (C-C motif) ligand 2 gene, *IFNL3* - interferon lambda 3 gene, *IL* - interleukin gene, *IL4R* - interleukin 4 receptor gene, N - number of deaths among tested patients

a - Multiple-sample test P

b – Log rank test P

Table S7. Statistical significance of differences in infection-related mortality evaluated by the Kaplan-Meier analysis for T helper cell cytokine genes in hemodialysis patients undergoing the 7-year prospective study

| Tested polymorphism | N | Major homozytes vs. heterozygotes vs. minor homozygotes^a^ | Dominant model of inheritance^b^ | Recessive model of inheritance^b^ | Additive model of inheritance^b^ |
| --- | --- | --- | --- | --- | --- |
| *CCL2* rs1024611 | 30 | AA vs. AG vs. GG  P = 0.343 | GG + AG vs. AA  P = 0.797 | GG vs. AG + AA  P = 0.356 | GG vs. AA  P = 0.393 |
| *IFNL3* rs8099917 | 33 | TT vs. GT vs. GG  P = 0.237 | GG + GT vs. TT  P = 0.346 | GG vs. GT + TT  P = 0.661 | GG vs. TT  P = 0.997 |
| *IFNL3* rs12979860 | 33 | CC vs. CT vs. TT  P = 0.296 | TT + CT vs. CC  P = 0.874 | TT vs. CT + CC P = 0.492 | TT vs. CC  P = 0.419 |
| *IL4R* rs1805015 | 34 | TT vs. CT vs. CC  P = 0.302 | CC + CT vs. TT  P = 0.299 | CC vs. CT + TT  P = 0.432 | CC vs. TT  P = 0.419 |
| *IL12A* rs568408 | 37 | GG vs. AG vs. AA P = 0.124 | AA + AG vs. GG P = 0.099 | AA vs. AG + GG  P = 0.185 | AA vs. GG  P = 0.074 |
| *IL12B* rs3212227 | 37 | AA vs. AC vs. CC  P = 0.277 | CC + AC vs. AA  P = 0.796 | CC vs. AC + AA  P = 0.019^c^ | CC vs. AA  P = 0.064 |
| *IL13* rs20541 | 34 | CC vs. CT vs. TT  NA | TT + CT vs. CC  P = 0.568 | TT vs. CT + CC  NA | TT vs. CC  NA |
| *IL18* rs360719 | 39 | TT vs. CT vs. CC  P = 0.366 | CC + CT vs. TT  P = 0.869 | CC vs. CT + TT  P = 0.487 | CC vs. TT  P = 0.387 |

Abbreviations: *CCL2* - chemokine (C-C motif) ligand 2 gene, *IFNL3* - interferon lambda 3 gene, *IL* - interleukin gene, *IL4R* - interleukin 4 receptor gene, N - number of deaths among tested patients

a - Multiple-sample test P

b – Log rank test P

c – There was only one patient with CC genotype. The P value in the Cox proportional model was 0.199.

Table S8. Statistical significance of differences in neoplasm-related mortality evaluated by the Kaplan-Meier analysis for T helper cell cytokine genes in hemodialysis patients undergoing the 7-year prospective study

| Tested polymorphism | N | Major homozytes vs. heterozygotes vs. minor homozygotes^a^ | Dominant model of inheritance^b^ | Recessive model of inheritance^b^ | Additive model of inheritance^b^ |
| --- | --- | --- | --- | --- | --- |
| *CCL2* rs1024611 | 24 | AA vs. AG vs. GG  NA | GG + AG vs. AA  P = 0.177 | GG vs. AG + AA  NA | GG vs. AA  NA |
| *IFNL3* rs8099917 | 21 | TT vs. GT vs. GG  P = 0.681 | GG + GT vs. TT  P = 0.936 | GG vs. GT + TT  P = 0.505 | GG vs. TT  P = 0.614 |
| *IFNL3* rs12979860 | 22 | CC vs. CT vs. TT  P = 0.638 | TT + CT vs. CC  P = 0.380 | TT vs. CT + CC P = 0.674 | TT vs. CC  P = 0.818 |
| *IL4R* rs1805015 | 27 | TT vs. CT vs. CC  P = 0.357 | CC + CT vs. TT  P = 0.166 | CC vs. CT + TT  P = 0.360 | CC vs. TT  P = 0.343 |
| *IL12A* rs568408 | 27 | GG vs. AG vs. AA P = 0.749 | AA + AG vs. GG P = 0.477 | AA vs. AG + GG  P = 0.697 | AA vs. GG  P = 0.755 |
| *IL12B* rs3212227 | 28 | AA vs. AC vs. CC  P = 0.179 | CC + AC vs. AA  P = 0.087 | CC vs. AC + AA  P = 0.470 | CC vs. AA  P = 0.393 |
| *IL13* rs20541 | 27 | CC vs. CT vs. TT  P = 0.650 | TT + CT vs. CC  P = 0.385 | TT vs. CT + CC P = 0.619 | TT vs. CC  P = 0.630 |
| *IL18* rs360719 | 30 | TT vs. CT vs. CC  P = 0.061 | CC + CT vs. TT  P = 0.147 | CC vs. CT + TT  P = 0.165 | CC vs. TT  P = 0.138 |

Abbreviations: *CCL2* - chemokine (C-C motif) ligand 2 gene, *IFNL3* - interferon lambda 3 gene, *IL* - interleukin gene, *IL4R* - interleukin 4 receptor gene, N - number of deaths among tested patients

a - Multiple-sample test P

b – Log rank test P

Table S9. Haplotype analysis of *IFNL3* polymorphic variants in patients who died on HD (cases) and those who survived on HD for 7 years (controls)

|  | | | |  |  |  |
| --- | --- | --- | --- | --- | --- | --- |
|  |  |  |  |  |  |  |
| Polymorphisms | Haplotypes | Frequency | Case, Control Frequencies | χ^2^ | p value | p_corr_ value^a^ |
| rs12979860_rs8099917 | CT | 0.623 | 0.619, 0.632 | 0.127 | 0.722 | 0.959 |
|  | TG | 0.198 | 0.210, 0.174 | 1.323 | 0.250 | 0.579 |
|  | TT | 0.172 | 0.165, 0.189 | 0.654 | 0.419 | 0.764 |
|  |  |  |  |  |  |  |
| ^a^ p value calculated using permutation test and a total of 1,000 permutations | | | |  |  |  |

Table S10. Epistatic interactions between genes in patients who died on HD and those who survived on HD for 7 years

| CHR1 | SNP1 | CHR2 | SNP2 | OR_INT | STAT | P |
| --- | --- | --- | --- | --- | --- | --- |
| 3 | rs568408 | 5 | rs20541 | 1.115 | 0.0868 | 0.7683 |
| 3 | rs568408 | 5 | rs3212227 | 0.672 | sty-80 | 0.2565 |
| 3 | rs568408 | 11 | rs360719 | 0.760 | 0.6709 | 0.4127 |
| 3 | rs568408 | 16 | rs1805015 | 0.780 | 0.4832 | 0.4870 |
| 3 | rs568408 | 17 | rs1024611 | 0.695 | 0.8982 | 0.3433 |
| 3 | rs568408 | 19 | rs12979860 | 0.801 | 0.5501 | 0.4583 |
| 3 | rs568408 | 19 | rs8099917 | 1.299 | 0.4257 | 0.5141 |
| 5 | rs20541 | 5 | rs3212227 | 1.199 | 0.3451 | 0.5569 |
| 5 | rs20541 | 11 | rs360719 | 1.133 | 0.1813 | 0.6703 |
| 5 | rs20541 | 16 | rs1805015 | 0.865 | 0.2301 | 0.6314 |
| 5 | rs20541 | 17 | rs1024611 | 0.919 | 0.0692 | 0.7925 |
| 5 | rs20541 | 19 | rs12979860 | 0.726 | 1.376 | 0.2408 |
| 5 | rs20541 | 19 | rs8099917 | 0.897 | 0.0888 | 0.7657 |
| 5 | rs3212227 | 11 | rs360719 | 0.691 | 1.212 | 0.2710 |
| 5 | rs3212227 | 16 | rs1805015 | 1.114 | 0.1001 | 0.7517 |
| 5 | rs3212227 | 17 | rs1024611 | 0.666 | 1.295 | 0.2552 |
| 5 | rs3212227 | 19 | rs12979860 | 0.943 | 0.0400 | 0.8414 |
| 5 | rs3212227 | 19 | rs8099917 | 0.713 | 0.837 | 0.3603 |
| 11 | rs360719 | 16 | rs1805015 | 0.724 | 1.044 | 0.3069 |
| 11 | rs360719 | 17 | rs1024611 | 1.612 | 2.147 | 0.1428 |
| 11 | rs360719 | 19 | rs12979860 | 0.898 | 0.1642 | 0.6854 |
| 11 | rs360719 | 19 | rs8099917 | 1.100 | 0.0939 | 0.7592 |
| 16 | rs1805015 | 17 | rs1024611 | 0.860 | 0.186 | 0.6662 |
| 16 | rs1805015 | 19 | rs12979860 | 1.093 | 0.1001 | 0.7517 |
| 16 | rs1805015 | 19 | rs8099917 | 1.346 | 0.5878 | 0.4433 |
| 17 | rs1024611 | 19 | rs12979860 | 0.948 | 0.0368 | 0.8479 |
| 17 | rs1024611 | 19 | rs8099917 | 0.834 | 0.3017 | 0.5828 |
| 19 | rs12979860 | 19 | rs8099917 | 1.547 | 1.519 | 0.2177 |

| CHR1 Chromosome of first SNP | |
| --- | --- |
| SNP1 Identifier for first SNP |  |
| CHR2 Chromosome of second SNP | |
| SNP2 Identifier for second SNP | |
| OR_INT Odds ratio for interaction | |
| STAT Chi-square statistic, 1df |  |
| P Asymptotic p-value |  |
